# Mesangial Cell-Binding Activity of Serum Immunoglobulin G in Patients with Lupus Nephritis

**DOI:** 10.1371/journal.pone.0101987

**Published:** 2014-07-09

**Authors:** Desmond Y. H. Yap, Susan Yung, Qing Zhang, Colin Tang, Tak Mao Chan

**Affiliations:** Division of Nephrology, Department of Medicine, Queen Mary Hospital, The University of Hong Kong, Hong Kong; Weizmann Institute of Science, Israel

## Abstract

In vitro data showed that immunoglobulin G (IgG) from patients with lupus nephritis (LN) could bind to cultured human mesangial cells (HMC). The clinical relevance of such binding was unknown. Binding of IgG and subclasses was measured in 189 serial serum samples from 23 patients with Class III/IV±V LN (48 during renal flares, 141 during low level disease activity (LLDA)). 64 patients with non-lupus glomerular diseases (NLGD) and 23 healthy individuals were used as controls. HMC-binding was measured with cellular ELISA and expressed as OD index. HMC-binding index of total IgG was 0.12±0.09, 0.36±0.25, 0.59±0.37 and 0.74±0.42 in healthy subjects, NLGD, LN patients during LLDA, and LN flares respectively (*P* = 0.046, LN flare vs. LLDA; *P*<0.001, for healthy controls or NLGD vs. LN during flare or LLDA). Binding of serum IgG_1_ to HMC was 0.05±0.05, 0.15±0.11, 0.41±0.38 and 0.55±0.40 for the corresponding groups respectively (*P* = 0.007, LN flare vs. remission; *P*<0.001, for healthy controls or NLGD vs. LN during flare or remission). IgG_2_, IgG_3_ and IgG_4_ from patients and controls did not show significant binding to HMC. Total IgG and IgG_1_ HMC-binding index correlated with anti-dsDNA level (r = 0.26 and 0.39 respectively, *P*<0.001 for both), and inversely with C3 (r = −0.17 and −0.45, *P*<0.05 for both). Sensitivity/specificity of total IgG or IgG_1_ binding to HMC in predicting renal flares were 81.3%/39.7% (ROC AUC 0.61, *P* = 0.03) and 83.8%/41.8% (AUC 0.63, *P* = 0.009) respectively. HMC-binding by IgG_1,_ but not total IgG, correlated with mesangial immune deposition in LN renal biopsies under electron microscopy. Our results showed that binding of serum total IgG and IgG_1_ in LN patients correlates with disease activity. The correlation between IgG_1_ HMC-binding and mesangial immune deposition suggests a potential pathogenic significance.

## Introduction

Lupus nephritis (LN) is a severe manifestation in patients with systemic lupus erythematosus (SLE), and is an important cause of renal failure in some racial groups such as Asians [1], . The treatments to date are based on non-selective immunosuppression. The pathogenic mechanisms are multifactorial and complex, but increased understanding of the pathogenic mechanisms could lead to improvements in disease activity monitoring and treatment. SLE is a prototype autoimmune disease and is characterized by the production of different autoantibodies, resulting in immune-mediated injury to various organs including the kidneys [3], [4]. The pathogenic importance of anti-dsDNA antibodies is exemplified by in vitro studies demonstrating their presence in renal eluates obtained from patients and mice with LN [5]–[7], and by clinical observations demonstrating a correlation between anti-dsDNA antibody titre and disease activity [8].

Mesangial cells have a central location in the glomerulus. Not only do mesangial cells provide structural support to adjacent capillary loops but it is well established that they also participate actively in disease mechanisms through the production of inflammatory and fibrotic growth factors [9], [10]. Immunoglobulin deposition in the mesangial area, mesangial cell proliferation, and increase in mesangial matrix are constant features in renal biopsies of active LN [3], [11]. Our group has previously reported that anti-dsDNA isolated from patients with LN can bind to human mesangial cells (HMC) and such binding correlates with clinical activity in selected LN patients and could contribute to intra-renal disease pathogenesis [12], [13]. We also observed a correlation between anti-dsDNA and total IgG levels. In this study, we investigated whether the binding activity of total serum IgG and its subclasses to HMC might have clinical correlations in patients with LN, which have implications on the use of such binding as a biomarker for disease monitoring and further exploration into its pathogenic importance.

## Materials and Methods

### Patients

This study and the consent procedures were approved by the Institutional Review Board of the University of Hong Kong/Hospital Authority Hong Kong West Cluster (HKU/HA HKW IRB). All included subjects have signed consent for the use of the serum samples in this study and the consent forms are kept in patients' case records. Patients attending follow-up at the SLE Clinic of Queen Mary Hospital, Hong Kong, with biopsy-proven Class III/IV±V LN and two or more episodes of renal flare during the period 2001 to 2013 were included. Histological findings of LN were reported in accordance with the International Society of Nephrology/Renal Pathology Society (ISN/RPS) 2003 classification [14], [15]. Standard treatment for active LN included corticosteroids combined with either cyclophosphamide or mycophenolate mofetil (MMF) as induction immunosuppression, followed by low-dose corticosteroids with either azathioprine or MMF as long-term maintenance immunosuppression. Disease activity was categorized as “active” or “low level disease activity” (LLDA) on the basis of both clinical and serologic parameters. “Active” disease had SLE Disease Activity Index (SLEDAI) score >10 with ≥4 points in the renal domain, and “low level disease activity” status was defined by SLEDAI score <4 with no points in the renal domain [16]. Patients with non-lupus glomerular diseases (NLGD) and healthy subjects (age- and sex-matched) were included as controls. The NLGD group included patients with IgA nephropathy, minimal change nephropathy, membranous nephropathy and ANCA-associated glomerulonephritis, and serum samples were obtained at presentation when the diagnoses were established by renal biopsy.

### Laboratory methods

Archived serum samples from LN patients collected at baseline (i.e. at initiation of induction therapy) then serially at 3-month intervals with informed consent were retrieved. Single serum samples were obtained from patients with non-lupus glomerular diseases and healthy subjects.

HMC-binding activity of IgG in serum samples was measured using a cellular ELISA as previously described [12]. Briefly, HMC were seeded into 96-well microtitre plates at a density of 10,000 cells/cm^2^. Cells were cultured in RPMI 1640 medium supplemented with 15% FCS and medium changed every 3 days. At 90% confluence HMC were growth arrested for 72 h, washed with PBS then fixed with 1% paraformaldehyde in PBS for 15 min. Cells were washed thrice with PBS in between steps, and all incubations were for 1 h at 37°C. HMC were blocked with 3% BSA followed by normal IgG (100 µg/ml) to block Fc receptor-mediated binding. HMC were incubated with serum samples (diluted 1∶100, 100 µl) in triplicate, then incubated with anti-human IgG F(ab) conjugated with alkaline phosphatase. Degree of IgG binding to HMC was detected by incubation with para-nitrophenol phosphate at room temperature and with optical density measurement at A_405/420_ when pre-established positive control sample showed an optical density of 1.5. The positive control was pooled serum from a patient with high HMC-binding activity. Seropositivity for HMC binding was denoted by results that exceed mean+3SD of results from healthy subjects. Circulating anti-dsDNA antibody titre was measured using a commercial ELISA (Microplate autoimmune anti-DNA quantitative ELISA) according to the manufacturer's instructions (BioRad, Hong Kong). Samples giving a value >60 IU/ml were considered positive. Kidney biopsies were performed within one week when renal flares were suspected clinically, and were reviewed by the same pathologist. The amount of mesangial deposits was semi-quantitated by electron microscopy (EM) in the following scale: 0 = no deposit; 1 = scanty deposits; 2 = moderate deposits; 3 = numerous deposits.

### Data analysis and statistics

Continuous variables were expressed as mean±SD and analyzed by student's t-test, unless otherwise specified. Categorical variable were expressed as frequencies and percentages, and analyzed with Chi-square test where appropriate. Correlation of HMC-binding activity with clinical parameters was assessed by the Spearman's method. The sensitivity/specificity, positive and negative predictive value (PPV and NPV) of HMC-binding activity in predicting renal flares was calculated, and the Area Under Curve (AUC) of the Receiver Operator Characteristics (ROC) curves was determined. Statistical analysis was performed by Graphpad Prism 5 (La Jolla, California, USA) and two-tailed *P values<0.05* was considered statistically significant.

## Results

### Patient characteristics

Twenty-three Chinese patients with biopsy-proven Class III/IV±V LN and who had experienced at least two episodes of renal flares during follow-up of 138.2±80.5 months were included (Table 1). A total of 276 serum samples were analyzed, with 189 samples from LN patients (48 during active renal flare and 141 during LLDA, 64 samples from patients with non-lupus glomerular diseases (NLGD), and 23 samples from healthy subjects respectively.

**Table 1:** Characteristics of 23 patients with Class III/IV±V lupus nephritis who had two or more episodes of renal flare during follow-up and included in the present study.

**Age (year)**	39.4±11.2
**Female/Male**	14/9
**Duration of follow up (month)**	138.2±80.5
**Previous immunosuppressive exposure**	
Prednisolone	23 (100%)
Cyclophosphamide	17 (73.9%)
Mycophenolate mofetil	18 (78.3%)
Azathioprine	19 (82.6%)
Calcineurin inhibitors	8 (34.8%)
**Laboratory parameters at first renal flare**	
Serum creatinine level (µmol/L)	111.8±56.4
Urine protein (g/D)	3.1±3.4
Anti-dsDNA level (iu/mL)	238.7±249.3
C3 level (mg/dL)	64.4±38.1

### HMC-binding activity of serum total IgG and its subclasses in LN patients

**Figure 1:**
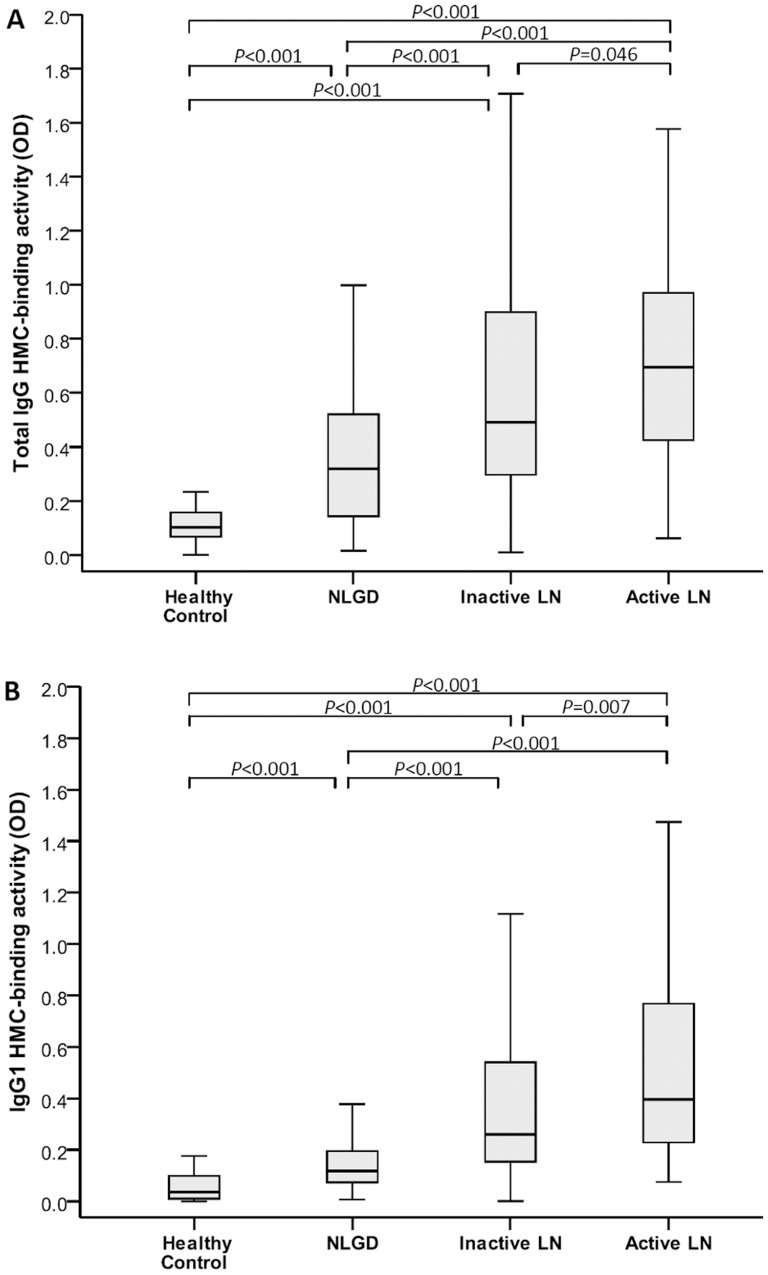
Mesangial cell-binding by (A) total IgG and (B) IgG_1_ in serum samples of patients with inactive or active lupus nephritis, non-lupus glomerular diseases, and patients with inactive or active lupus nephritis, non-lupus glomerular diseases, and healthy controls.

Binding index of serum total IgG to HMC was 0.12±0.09, 0.36±0.25, 0.59±0.37 and 0.74±0.42 for healthy controls, NLGD, LN patients during LLDA, and active LN respectively (*P* = 0.046, LN flare vs. LLDA; *P*<0.001, healthy controls or NLGD vs. active or inactive LN; *P*<0.001, NLGD vs. active or inactive LN) (Figure 1, A). Binding index of serum IgG_1_ to HMC was 0.05±0.05, 0.15±0.11, 0.41±0.38 and 0.55±0.40 for healthy controls, NLGD, LN patients in LLDA, and active LN respectively (*P* = 0.007, LN flare vs. LLDA; *P*<0.001, healthy controls or NLGD vs. active or inactive LN; *P*<0.001, NLGD vs. active or inactive LN) (Figure 1, B). There was no significant binding of serum IgG_2_, IgG_3_ or IgG_4_ to HMC, which also did not vary between healthy controls, NLGD patients, and LN patients during flare or LLDA.

### HMC binding by serum IgG and IgG_1_ and clinical parameters

**Figure 2:**
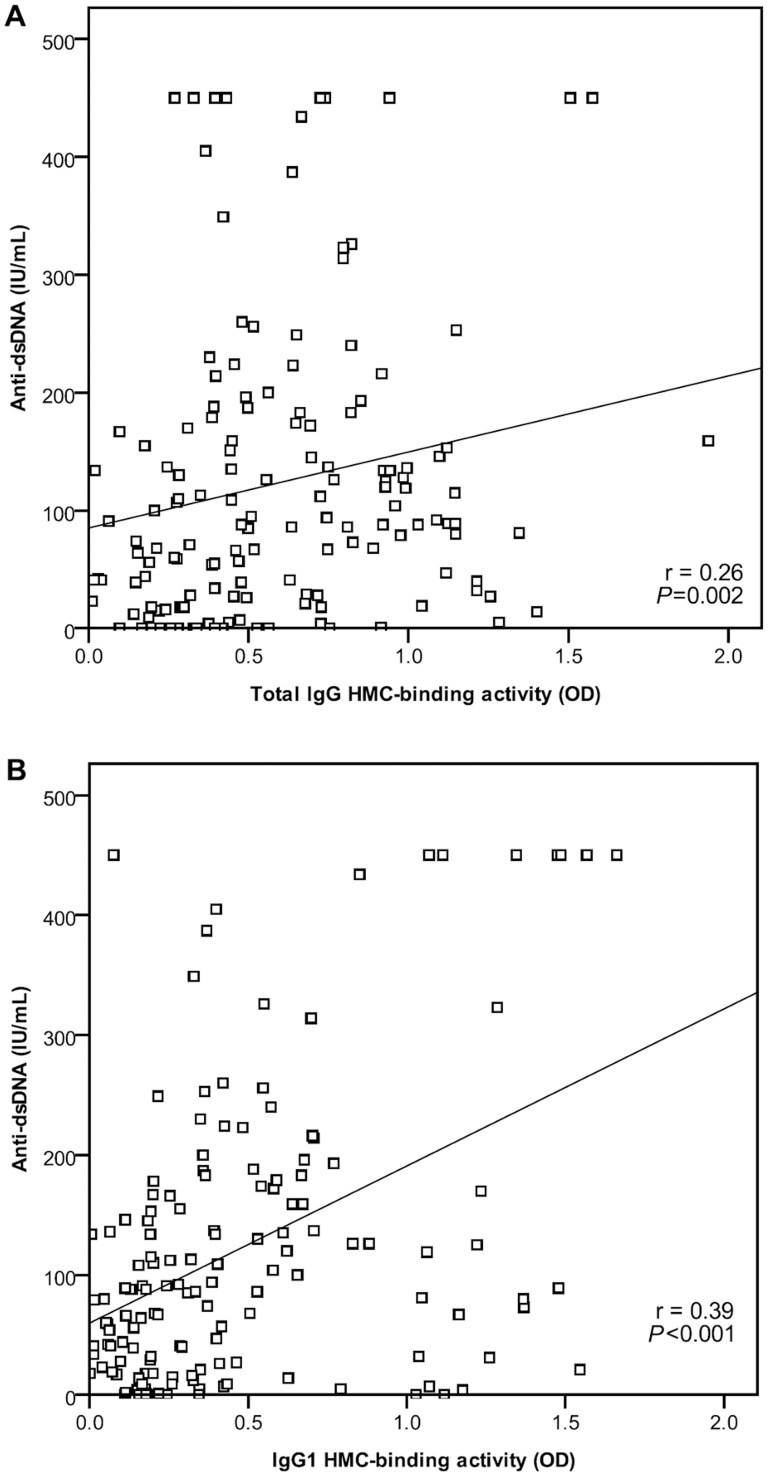
Correlation between mesangial cell-binding by (A) total IgG and (B) IgG_1_ with anti-dsDNA level in 23 patients with Class III/IV±V lupus nephritis.

**Figure 3:**
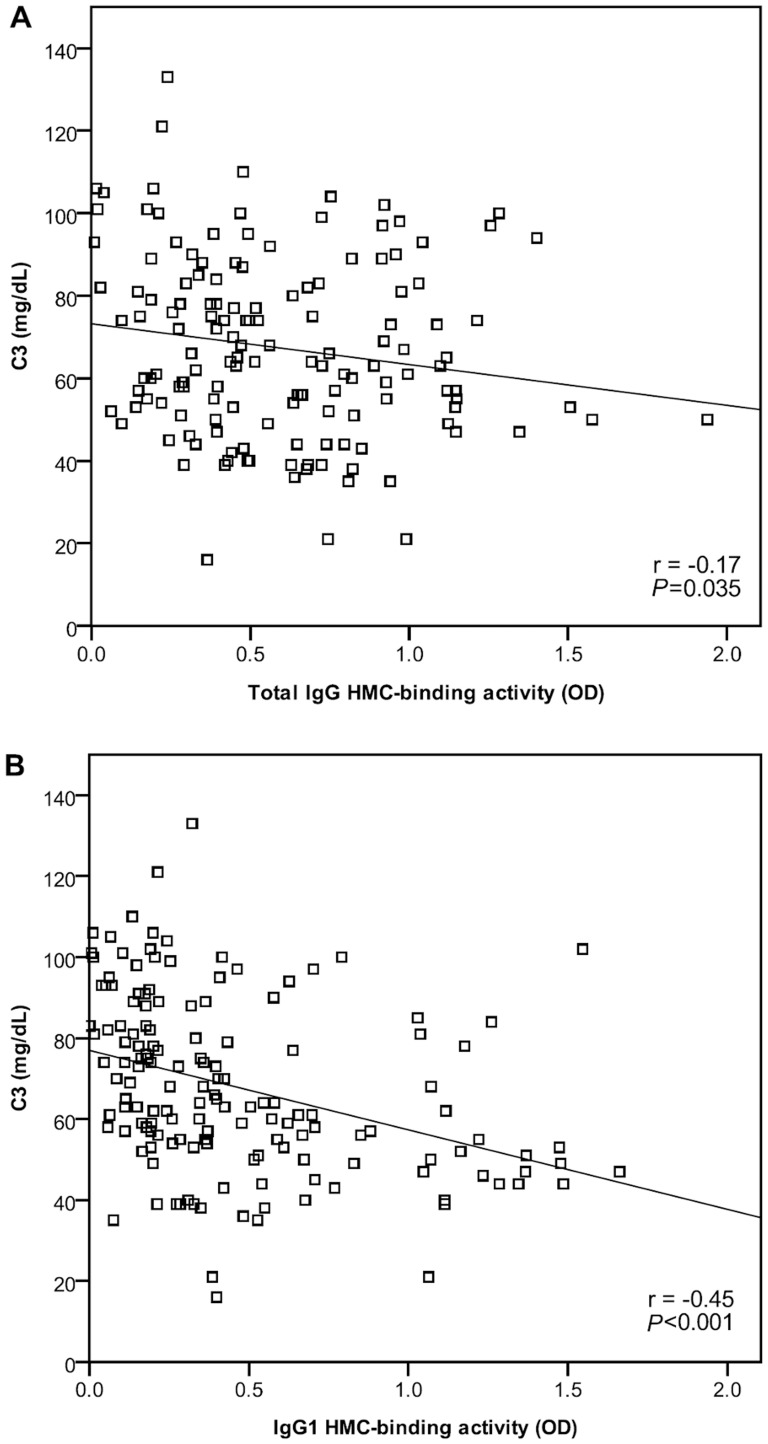
Negative correlation between mesangial cell-binding by (A) total IgG and (B) IgG_1_ with C3 level in 23 patients with Class III/IV±V lupus nephritis.

Total serum IgG and serum IgG_1_ HMC-binding index both correlated positively with anti-dsDNA levels (r = 0.26 and 0.39 respectively, *P*<0.001 for both) (Figure 2, A&B). In contrast, both showed a negative correlation with C3 levels (r = −0.17 and −0.45 respectively, *P*<0.05 for both) (Figure 3, A&B). HMC-binding by IgG and IgG_1_ was not related to the level of serum creatinine, serum albumin, or proteinuria at the time of blood sample collection (*P* = 0.735, 0.546 and 0.700 respectively for total IgG HMC-binding; *P* = 0.628, 0.443 and 0.170 for IgG_1_ HMC-binding).

**Figure 4:**
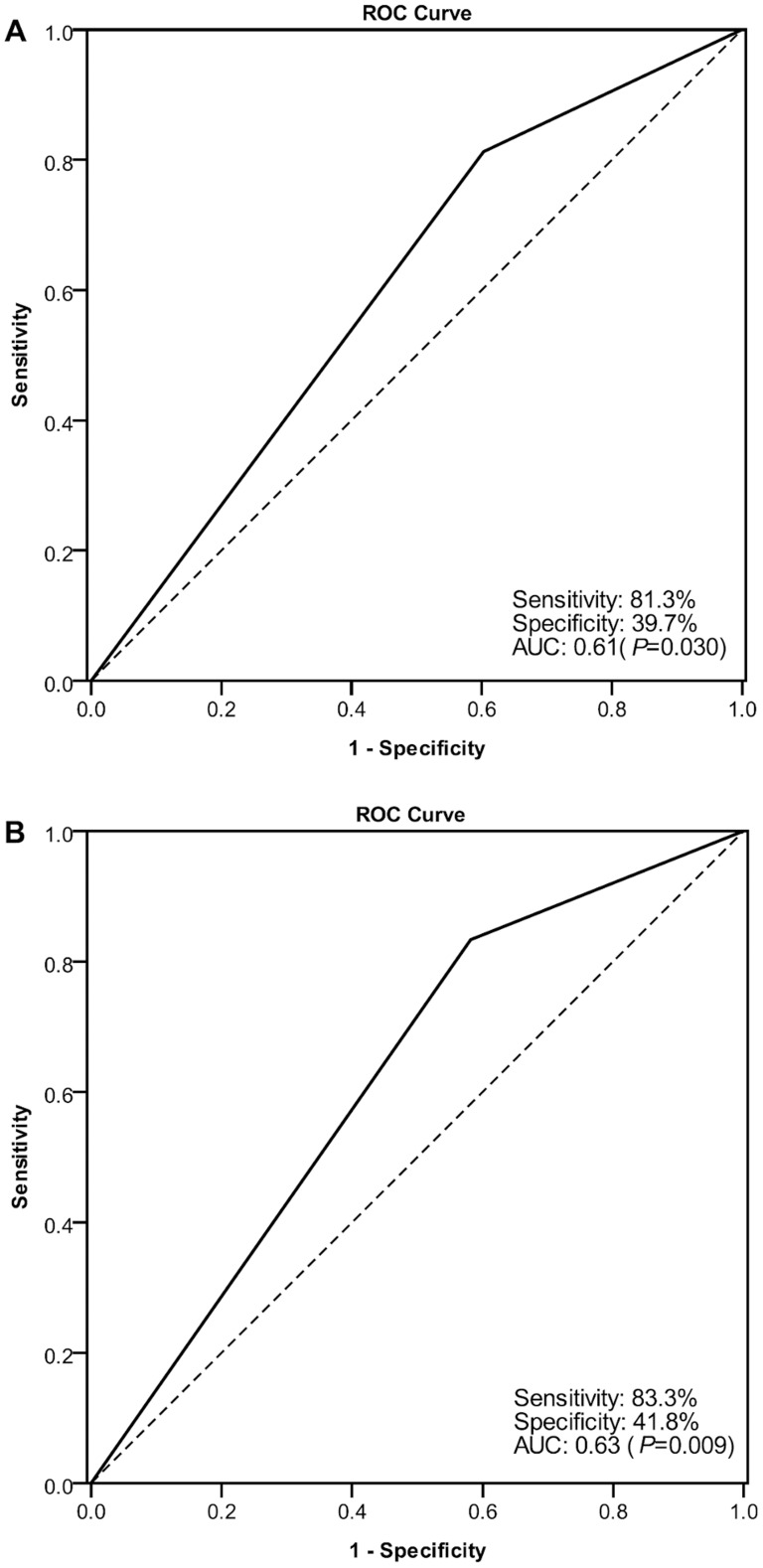
Receiver Operator Characteristics (ROC) curve for sensitivity/specificity in renal flare prediction using the level of mesangial cell-binding by (A) total IgG and (B) IgG_1_ in serum samples of 23 patients with Class III/IV±V lupus nephritis.

Overall sensitivity/specificity of total IgG or IgG_1_ HMC-binding index in the prediction of renal flares (with or without systemic flares) was 81.3%/39.7% (AUC 0.61, *P* = 0.030) and 83.8%/41.8% (AUC 0.63, *P* = 0.009) respectively (Figure 4, A&B). The overall PPV and NPV of total IgG or IgG1 HMC-binding index in prediction of renal flares (with or without systemic flares) were 31.5%/86.2% and 32.8%/88.1% respectively. The sensitivity/specificity of total IgG or IgG_1_ HMC-binding index to predict renal flares with concomitant systemic flares were 80.0%/35.1% (PPV: 12.1%; NPV: 94.1%) and 83.3%/36.1% (PPV: 12.7%; NPV: 95.1%), and 79.6%/38.6% (PPV: 28.2%; NPV: 86.2%) and 83.3%/40.8% (PPV: 28.7%; NPV: 89.6%) for renal flares without systemic flares. Two patients (8.7%) showed positive total IgG and IgG_1_ binding to HMC during active renal flare when their anti-dsDNA and C3 levels remained within normal limits. Seropositivity of HMC-binding IgG_1_ precedes renal flares by 43.8±62.9 days.

### HMC binding by serum IgG and IgG_1_ and mesangial immune deposition assessed by electron microscopy

Total IgG HMC-binding seropositivity was not related to the degree of mesangial immune deposition (*P* = 0.733). In contrast, patients seropositive for IgG_1_ HMC-binding were more likely to show increased mesangial immune deposition at grades 2 or 3 (i.e. moderate to numerous) (*P* = 0.016). HMC-binding index of IgG_1_, but not total IgG, also correlated positively with the mesangial deposition score (r = 0.776 and *P*<0.001 for IgG_1_; r = 0.263 and *P* = 0.363 for total IgG).

## Discussion

SLE is characterized by the production of various autoantibodies [4]. Previous studies have reported that different autoantibodies can bind to different renal components including podocytes, mesangial cells, endothelial cells, and renal tubular epithelial cells, and it has been speculated that such binding could have a pathogenic role in immune-mediated kidney injury [10], [12], [13], [17]–[19]. Mesangial cells are located strategically at the centre of glomeruli and are juxtaposed to the capillary loops [9]. Mesangial immunoglobulin deposition and mesangial cell proliferation are cardinal histological features in LN [3], [11]. Our previous investigations showed that anti-dsDNA antibodies from patients with LN could bind to HMC and trigger cellular responses involved in inflammation and fibrosis, and that such binding correlated with total serum IgG level (10,12). The present study sought to investigate the potential clinical correlations of the in vitro findings.

Our results showed a clear relationship between disease activity and HMC-binding by total serum IgG and IgG_1_ in LN, and the degree of binding was significantly increased in LN compared with healthy subjects and patients with non-lupus glomerular diseases. Also, the positive correlation of IgG HMC-binding with anti-dsDNA level, and negative relationship with C3 level, prompted us to investigate whether HMC-binding index might serve as a biomarker for disease activity monitoring. In this context, previous studies have also found that active LN patients had significantly stronger IgG binding to isolated rat glomeruli in an *in vitro* assay when compared to SLE patients without nephritis [20]. That HMC-binding index of total IgG or IgG_1_ was not related to serum creatinine, serum albumin, or proteinuria was not a disadvantage since these clinical parameters represent a summative outcome of both active disease and prior chronic damage and are also subject to modulating factors distinct from the lupus disease process such as hypertensive renal damage. Conventional serological parameters C3 and anti-dsDNA levels have been reported to show sensitivity and specificity of 49–79% and 51–74% respectively in the detection of disease flares [21]–[27]. The present results from samples collected serially in LN patients showed that in the majority of cases increased HMC-binding by IgG and IgG_1_ was associated with increased disease activity, so that these parameters had sensitivities of over 80% in the predication of renal flares. However, seropositivity for HMC-binding by itself could be present in patients during remission and thus was non-specific for active disease. Notwithstanding its lack of specificity, assessment of HMC-binding index may be of value in the small proportion of patients in whom conventional serological parameters such as anti-DNA and C3 levels do not correlate with disease activity, as was demonstrated in two of the 23 patients studied, when both anti-DNA and C3 were still within the normal range at disease flare but serum HMC-binding IgG was positive.

The present results also have implications on pathogenic mechanisms in LN. Among the different IgG subclasses from LN patients tested, only IgG_1_ showed significant binding to HMC. Furthermore, HMC-binding by IgG_1_ correlated with clinical disease activity and also the degree of mesangial immune deposition as assessed by electron microscopy. In this context, previous studies have suggested that IgG_1_ might be more pathogenic compared with other IgG subclasses in LN, attributed to its ability to fix complements [28], [29]. Further studies are required to investigate the downstream cellular processes that follow the binding of HMC by IgG_1_, and whether intervention or interruption of such binding could present a novel therapeutic approach.

## Conclusions

The degree of mesangial cell binding by circulating IgG and IgG_1_ in serum samples of patients with LN correlates with disease activity, and thus may complement anti-dsDNA and C3 as biomarkers for disease monitoring. Its relationship with mesangial immunoglobulin deposition in LN kidney tissue also suggests a pathogenic role.
